# Physicochemical property distributions for accurate and rapid pairwise protein homology detection

**DOI:** 10.1186/1471-2105-11-145

**Published:** 2010-03-19

**Authors:** Bobbie-Jo M Webb-Robertson, Kyle G Ratuiste, Christopher S Oehmen

**Affiliations:** 1 Computational Biology and Bioinformatics, Pacific Northwest National Laboratory, Richland, WA 99352, USA; 2 Department of Chemistry, Gonzaga University, Spokane, WA 99258, USA

## Abstract

**Background:**

The challenge of remote homology detection is that many evolutionarily related sequences have very little similarity at the amino acid level. Kernel-based discriminative methods, such as support vector machines (SVMs), that use vector representations of sequences derived from sequence properties have been shown to have superior accuracy when compared to traditional approaches for the task of remote homology detection.

**Results:**

We introduce a new method for feature vector representation based on the physicochemical properties of the primary protein sequence. A distribution of physicochemical property scores are assembled from 4-mers of the sequence and normalized based on the null distribution of the property over all possible 4-mers. With this approach there is little computational cost associated with the transformation of the protein into feature space, and overall performance in terms of remote homology detection is comparable with current state-of-the-art methods. We demonstrate that the features can be used for the task of pairwise remote homology detection with improved accuracy versus sequence-based methods such as BLAST and other feature-based methods of similar computational cost.

**Conclusions:**

A protein feature method based on physicochemical properties is a viable approach for extracting features in a computationally inexpensive manner while retaining the sensitivity of SVM protein homology detection. Furthermore, identifying features that can be used for generic pairwise homology detection in lieu of family-based homology detection is important for applications such as large database searches and comparative genomics.

## Background

A central problem in computational biology is the task of identifying distantly related evolutionary ancestors, *i.e*., remote homlogs from primary sequence. Currently, ~39% of the over 6.5 million proteins in the non-redundant database (nr_march_2008) remain simply as hypothetical, conserved hypothetical, or unknown. With the continued exponential growth of the sequence databases, improvement in the computational annotation of sequences is a necessity.

In recent years, much of the research in the area of remote homology detection has focused on the use of machine learning algorithms, largely support vector machines (SVMs) to build protein family centric predictive models leading to a large number of approaches [1-15]. The overall ability of these methods to identify homologs is based on the features used to encode the protein sequences. Many early approaches to feature generation for protein sequences used amino acid similarity metrics to a basis set [4,8] or the frequency of specific patterns [3,16,17]. Further improvements in accuracy were achieved by accounting for additional information such as motif order or the likelihood of the occurrence of a motif [5,18]. Methods that are profile-based are to date the most accurate, achieving average area under the curve (AUC) for a receiver operating characteristic (ROC) curve value of ~0.98 on the standard SCOP 1.53 benchmark dataset [14]. Other higher accuracy approaches use latent semantic analysis or recurrence quantification. Additionally, methods based on network propagation have been developed to compare sequences to a database [19-23], but at a significant computational cost. For algorithms that are not as computationally demanding in the feature generation stage the average AUC values typically range from ~0.87 to ~0.9.

In bioinformatics, the most common application of homology detection is searching databases for related sequences through pairwise comparisons, most commonly BLAST [24] or PSI-BLAST [25]. To compete with these popular heuristic-based sequence methods new pairwise algorithms must be both simple and computationally friendly. Leslie et al., [3] demonstrated that kernel matrices can be used directly for pairwise comparisons. When compared to BLAST a string kernel was able to identify homologous relationships in SCOP 1.53 with a global AUC of ~0.70 at the superfamily level in comparison to ~0.66 for BLAST, a modest improvement.

We present a computationally streamlined implementation of SVM homology detection based on physicochemical distributions (SVM-PCD). These feature vectors have low computational cost by using physicochemical properties of amino acids based on the Amino Acid index (AAIndex) [26] in lieu of evolutionary information. Feature generation is based on the normalized distribution of the average AAIndex value over all sequential 4-mers in the sequence. We show that this new feature representation performs similarly or better than current family-based classification methods with a significant decrease in computation time. Most notably, we demonstrate with direct evaluation of similarity across each AAindex for a protein that pairwise homology detection can be performed with improved accuracy over methods such as BLAST and Smith-Waterman [27].

## Methods

### Generation and selection of null distributions from the AAindex

The goal of protein remote homology detection is to accurately classify protein sequences based on evolutionary relationships with the end goal of annotating new sequences of unknown structure and function. We present a new method that uses the average physicochemical property values associated with all 4-mers to transform a protein sequence into a series of probability distributions that can be used to define an accurate discriminative function of protein homology.

The Amino Acid index (AAindex) is a database of numerical values, where each number represents a specific physicochemical or biochemical property of an amino acid or pair of amino acids. The latest version of the database (version 9) is separated into three parts: AAindex1, AAindex2 and AAindex3. AAindex1 has 544 properties associated with each of the 20 amino acids, AAindex2 contains 94 amino acid substitution matrices, and AAindex3 contains 47 amino acid contact potential matrices. For the purpose of protein transformation, the matrices were not used, leaving the 544 amino acid properties (*i.e*., indices) as potential features. Of the 544 indices, 13 had incomplete data or an over-representation of zeros, and were removed. Thus 531 indices were evaluated for potential use in the protein transformation step.

**Figure 1:**
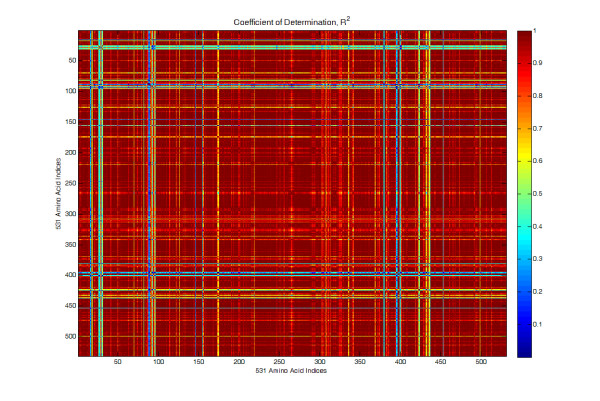
**Correlation between each of the 531 AA indices after transformation to discrete probability distributions**.

There is only moderate correlation between AAindex properties, however there is considerable correlation when considering all possible 4-mers, where each 4-mer is the average of the physicochemical property values of the corresponding amino acids. The choice of selecting amino acids in sets of 4 was based on prior work by Yang *et al*., [9] where a 4-mer was found to work well, as well as prior work by Leslie *et al*. [2,3], which found 4-mers to work well for string kernels based on un-gapped sequences of amino acids. To determine which of the 531 indices would be used to derive protein distribution features, each of the indices was transformed into a theoretical distribution associated with the values of the 160,000 possible 4-mers (20^4^). For each index the 160,000 average value of all possible 4-mers was computed and then transformed into a discrete empirical distribution. Correlated indices were identified by comparing each discrete probability distribution using a Pearson correlation coefficient of determination (*R*^2^), Figure 1. Three different subsets of indices were identified for evaluation: 1) 61 indices that have an *R*^2 ^value less than 0.99, 2) 181 indices that have an *R*^2 ^value less than 0.999, and 3) all indices. For example, the subset for which the *R*^2 ^value is less than 0.99 was selected by simply marching through the indices in order, e.g., if AAindex 4 and AAindex 20 were correlated at 0.995, the first index (AAindex 4) was kept.

### Protein sequence transformation into feature space

**Figure 2:**
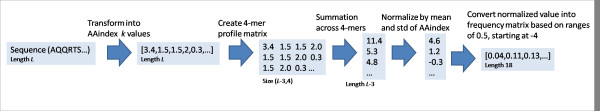
**Schematic of the process of feature generation for a single sequence**.

For a query sequence, the transformation to a distribution was computed by normalizing each of the 4-mer average index values to the mean and standard deviation of the theoretical values computed as described in the previous section and then binning the values into a discrete density distribution. Figure 2 gives a schematic of the overall process. In the first step the protein sequence of length *L *is transformed from the 20 amino acid letter code to a set of numerical values associated with the index being used also of length *L*. The average across all 4-mers was taken to create a new numerical vector that is of length *L*-3. Each of these values was then normalized to the mean and standard deviation of the theoretical values associated with the index,(1)

where *v*_*ijk *_is the value obtained for the i-th index on the j-th sequence for the k-th 4-mer, μ_*i *_and σ_*i *_are the mean and standard deviation of the index under consideration. These normalized values were then transformed into a discrete distribution of 18 frequency values, where each value represents a range of 0.5σ, *i.e*., the first bin is all values less than -4, the second bin is all values between -4 and -3.5, and so forth.

### Benchmark Dataset

A standard benchmark dataset is SCOP 1.53, used extensively for benchmarking and evaluating new SVM-based protein family discriminative algorithms. SCOP 1.53 consists of 4352 protein sequences, which collectively cover 560 protein superfamilies (a common level of SCOP hierarchy for defining homology). From this collection of data positive and negative training sets have been derived for the 54 superfamilies with the most members, described in detail by Liao and Noble [4]. The training and test set definitions are available at http://noble.gs.washington.edu/proj/svm-pairwise/. In addition, this dataset contains classifications on all proteins at the fold, superfamily and family levels, which can be used to assess accuracy of all pairwise comparisons at each evolutionary level.

### ROC Analyses

A ROC curve is a graphical representation of the false positive rate (FPR) versus true positive rate (TPR). A perfect classifier would have a TPR of one at a FPR of 0, and likewise a TPR of one at a FPR of 1. The AUC is then one. A random classifier would return essentially the same TPR value for each FPR value, creating a diagonal line as the plot and an AUC of 0.5. This standard approach was used when performing only one comparison, such as homologs versus non-homologs, *i.e*., the pairwise task. For a protein family-based analysis, sets of training and test sequences were selected for each of the 54 superfamilies (described above). A separate SVM was trained and tested and a single AUC computed for each family. This process was repeated for all 54 superfamilies, so that each family had a corresponding AUC value [4]. Thus, the final analysis of the entire family-based approach was the AUC versus the number of families that achieved a particular AUC value or better. The overall performance of each of the SVM methods evaluated was summarized by the mean of all the 54 AUC values computed (Additional File 1).

### Statistical Software

All feature vectors were generated in MatLab^® ^R2009b and exported as text files in GIST format. All SVM classifiers were generated and tested using the GIST SVM software http://www.bioinformatics.ubc.ca/gist/[28]. All default parameters were used with the exception that the kernel function was defined as either a quadratic or a radial basis function. The ROC Analyses were performed in MatLab^® ^using functional available through the Statistics Toolbox.

## Results and Discussion

**Figure 3:**
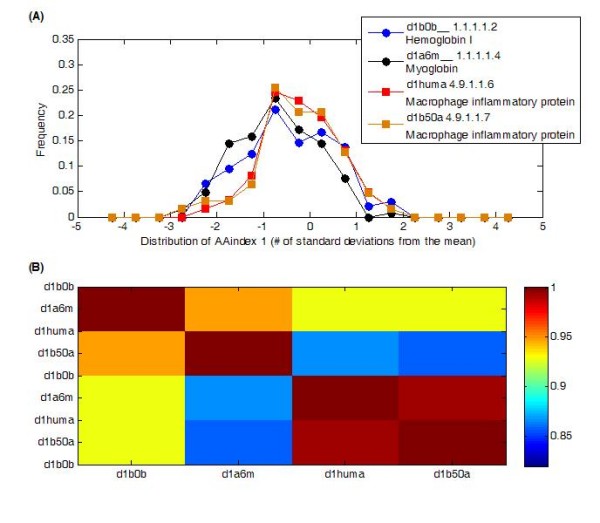
**Comparisons of two sets of homologous pairs by distribution and correlation**. (A) The discrete distributions or a pair of globin homologs versus a pair of homologous macrophage inflammatory proteins shows a clear similarity for the related pairs. (B) The correlation of the distribution in (a) between related pairs is evident, yielding correlations of ~0.95 and ~0.99 for each homologous pair, respectively.

The basic assumption of the physicochemical distribution approach is that proteins that are homologous will deviate in a similar manner from the null distribution generated from all possible 4-mers. The discrete distribution of a protein for a single AAindex is represented by frequency values across 18 bins associated with the number of standard deviations from the mean each of the observed values is, Eq. 1. Figure 3A gives these frequency values for two distinct homologous pairs, two that are from the globin-like family and the other two are macrophage inflammatory proteins (MIPs) for the first AAindex, Alpha-CH chemical shifts [29]. Clearly the related pairs have more similar distributions, which are also reflected in their correlation, Figure 3B. For this particular index the homologous pairs have correlation of ~0.95 and ~0.99 for the globins and MIPs, respectively. In comparison the correlations across these families range from ~0.86 to ~0.93. In this study we evaluated how well these distributions, concatenated into a single feature vector, can differentially identify homologous pairs.

In order to establish the PCD vectorization approach as comparable to other methods to identify homologous relationships between proteins a traditional family-based analysis was undertaken on the SCOP 1.53 dataset in a comparable fashion to many prior SVM-based protein family classification methods [1-15]. This was also performed to determine if all 531 AAindices are needed or if one of the subsets would be adequate. Three datasets were considered, each consisting of *k *AAindices by the 18-bin distribution, or equally k*18 variables; all 531 (9558 variables), 181 with *R*^2 ^less than 0.999 (3258 variables) and 61 with *R*^2 ^less than 0.99 (1098 variables); PCD(531), PCD(181), and PCD(61), respectively. For training and testing the SVM, no feature selection was performed to select the "best" AAindices for a particular sequence or the "best" parameters for the SVM since we are interested in the general robustness of the features. Prior work had tuned both the features and the SVM parameters to each specific family [9]. However, both a quadratic and RBF kernels were evaluated for each family to determine the most appropriate kernel transformation for the data associated with each family. For PCD (531) 34 families used the RBF kernel and 20 used the quadratic kernel. These values were 30 and 24 for the RBF and quadratic kernels, respectively, for PCD(181) and they were 33 and 21 for the RBF and quadratic kernels for PCD(61), respectively. Overall, SVM-PCD achieved an average AUC over the 54 families of 0.902, 0.902, and 0.906 on PCD(531), PCD(181) and PCD(61), respectively, which was better than some and worse than others. However, in the cases where SVM-PCD does not achieve as high in terms of accuracy it is dramatically faster in terms of vectorization speed. The results of SVM-PCD in comparison to other algorithms is in Additional File 1. This exercise in the family-based comparison demonstrated that SVM-PCD is a comparable method in terms of accuracy to approaches such as SVM-RQA and better than others such as SVM-LA. Thus, this vectorization approach is valid for implementation into a pairwise algorithm. In addition, this analysis demonstrated that no gain in accuracy is achieved beyond PCD(61) and thus an even smaller vectorization footprint from the full AAindex can be carried forward into the pairwise homology analysis.

### Pairwise homology analyses

The family-based ROC analysis showed that probability distributions based on physicochemical properties can be used to train a classifier to separate proteins by superfamily with similar accuracy as the current state-of-the-art methods. However, the family-based approaches do not have wide applicability because they require that an adequate number of proteins are known to be associated with a family in order to train a classifier. Traditional sequence-based analyses, such as BLAST do not have this requirement because they compare the sequences in a pairwise manner. To evaluate the generic nature of physicochemical property distributions for pairwise homology detection, all 4352 protein sequences in the SCOP 1.53 benchmark database can be compared against one another and the performance of the approach evaluated in a global manner with a ROC analysis.

The family-based analysis did not improve by including amino acid indices that had a correlation of greater than 0.99. Thus, only the set of indices with correlation values less than 0.99 was used to transform a protein *R*^(*j*) ^into the associated feature space (Figure 2), resulting in a vector of length 1098 (61 indices by 18 frequencies), Φ_*PC*_(*R*^(*j*)^). The kernel for two sequences *R*^(*x*) ^and *R*^(*y*) ^is the inner product:

This kernel gives a measure of similarity between the two sequences in feature space, in essence the more similar the distributions the larger the kernel value. In a similar manner to Leslie et al. [3], this kernel is used to generate a distance measure between two proteins. The kernel is first normalized to unity for identical vectors:

The distance between *R*^(*x*) ^and *R*^(*y*) ^is then

The performance of the distance measure *D*_*PC *_was evaluated at the fold, superfamily, and family levels and defined by the SCOP hierarchy [30]. Here a single ROC curve can be computed using knowledge on true and false homologous pairs. There are 156842, 98417 and 77870 true homologs and 9315286, 9373711 and 9394258 non-homologs at the fold, superfamily and family levels, respectively. The distance measure *D*_*PC *_performs as well or better than AUC values for the Smith-Waterman (SW), BLAST and Mismatch algorithms as described by Leslie et al., [3] for the task of pairwise protein homology detection (Table 1).

**Table 1:** AUC values for distance matrices of Mismatch and PCD versus common sequence comparison algorithms

	SW	BLAST	Mismatch (*k *= 4, *m *= 1)	*D*_*PC *_(PCD)
Fold	0.713	0.619	0.623	0.681
Superfamily	0.679	0.660	0.704	0.757
Family	0.820	0.737	0.784	0.792

PSI-BLAST is also a popular approach to remote homology detection, but is not truly a pairwise comparison algorithm, but a profile-based algorithm, *i.e*., it cannot determine homology without first searching a database to build a profile. This is the likely reason it was not included in prior work in comparing kernel distance matrices to sequence-based homology algorithms [3]. BLAST, Smith-Waterman, and the kernel approaches can take the *N *sequence and yield a set of *N *× *N *pairwise relationship scores independent from any other sequence information. For comparative purposes PSI-BLAST was run using the NCBI publicly available software and allowed to build the profile for a query by searching against the NR database for up to 20 iterations [31] and not surprisingly it performed somewhat better than the other approaches with an AUC of ~8 and ~0.85 at the superfamily and family levels respectively. However, the accuracy and computational speed of PSI-BLAST is related to the number of iterations and the size of the database used to generate the profile, which increases the computation time to weeks versus minutes for the methods in Table 1 to perform the same *N *× *N *comparison in respect to a single processor. PSI-BLAST is a great option for a small numbers of queries, but for large comparisons across databases such as NR to annotate new genomes BLAST is still the method used and thus the PCD approach would offer an alternative for they types of tasks.

The primary caveats with the current PCD approach and other kernel-based homology detection algorithms are associated with accuracy and usability. Although these methods are slightly better than heuristic-based approaches such as BLAST, they are not quite good enough to warrant the investment to modify current pipelines that use BLAST. Methods that take into account amino acid order in the vectorization step yield improved results in the family-based analyses, but would dramatically increase the computational cost. Fast approaches to integrate amino acid order into PCD features, as well as combining vectors of the kernel to train a SVM to classify protein pairs as homologous or non-homologous [31], is a topic of future work. In respect to usability, the only output is a score. Many users find value in evaluating the actual alignment produced. Future work would also include integrating the SVM-based homology algorithm with more advanced alignment algorithms, such as those that use centroids [32], to give the most probabilistically correct alignment information.

### Computational efficiency

The computational cost of transforming proteins into a vectorized form is often a significant barrier preventing widespread acceptance of new methods. When applying SVM methods to the pairwise homology problem, the bulk of the computational cost is in the vectorization of the query sequence, *i.e*., transforming the protein sequence into a fixed length vector. Furthermore, methods that require a basis set of proteins against which to derive feature scores (e.g., SVM-Pairwise and SVM-BALSA) [4,8] are targeted, meaning that feature space is directly tied to the families in the basis set, and thus are not well suited to the generic pairwise problem.

The top-performing family-based methods are also the most computationally expensive and require multiple complex steps to arrive at the final vector. By contrast, utilizing a string-based kernel [3] or AAindex-base string kernel, such as in the SVM-PCD method, requires much more simple calculations and hence a much reduced run-time. To illustrate the magnitude of this difference, Hochreiter [33] reported a run-time of 550 *hours *for local-alignment-based method on a particular benchmark of 20,000 sequences vs. a run-time of 380 *seconds *for a Mismatch kernel method on the same benchmark. With five orders-of-magnitude faster run time, the mismatch-type methods are ideal candidates for a generic pairwise implementation.

**Figure 4:**
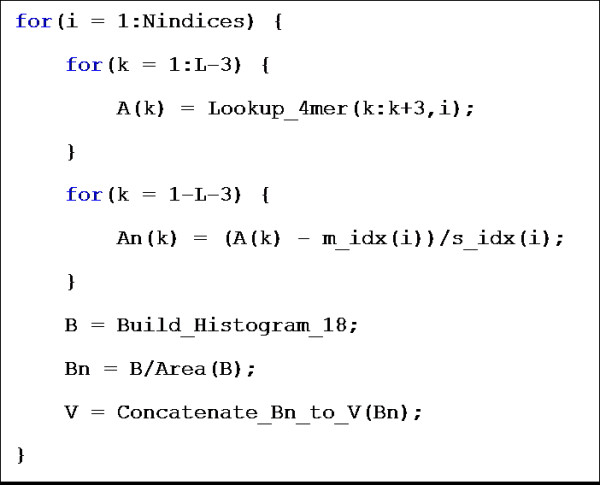
**Pseudocode for generation of a PCD vector from query sequence (length *L*) over *Nindices *AAindices**.

To demonstrate the simplicity of the PCD vectorization process, Figure 4 gives pseudocode for the calculation of the vector of a single query protein of length *L*. In the general case there are *N *indices selected from the AAindex and each has a lookup table of values associated with each possible 4-mer, which is used in the 'Lookup_4 mer' function. In addition, each AAindex has a mean (*m_idx*) and standard deviation (*s_idx*) value that are stored in a vector of length *NIndices*. Building the histogram is simply binning the normalized values based on a sliding window of size 0.5, starting at -4, and is represented as '*Build_Histogram_18*' function and is a quick and simple computation. Once the histogram is derived for an index it is simply concatenated to the ones already computed.

## Conclusions

We have presented a new approach to use physicochemical properties via the AAIndex to transform protein sequences into vector representation in a simple and computationally efficient manner. This new method, PCD, was evaluated using the common machine learning SVM approach of classifying proteins into predefined families. Our SVM-PCD method performed nearly as well as the computationally expensive SVM-RQA, which also uses physicochemical properties.

PCD is similar to string kernel methods in respect to computational costs and scaling. PCD was compared in a pairwise manner against the best string kernel presented by Leslie et al. (2004), (4,1)-Mismatch where 4 is the length of the k-mer and 1 is the number of allowed mis-matches. ROC analyses showed that our physicochemical property distributions offered an advantage over simple string comparisons for the identification of homologs at the fold, superfamily and family levels.

## Authors' contributions

BMW developed the mathematical method, performed statistical analyses, and wrote the manuscript. KGR helped to develop and evaluate feature generation methods. CSO performed computational comparisons and wrote the manuscript. All authors read and approved the final version of the manuscript.

## Supplementary Material

Additional file 1 **Table S1**. The average of the 54 AUC scores across multiple algorithms Click here for file
