# Supplementary material for: Physicochemical property distributions for accurate and rapid pairwise protein homology detection
Source: BMC Bioinformatics. 2010 Mar 19;11:145. doi: 10.1186/1471-2105-11-145 (PMC2851606; doi:10.1186/1471-2105-11-145)
Supplement: Additional file 1 — Table S1. The average of the 54 AUC scores across multiple algorithms [file 1471-2105-11-145-S1.DOC]

## Table S1 – The average of the 54 AUC scores across multiple algorithms

| Algorithm | Mean AUC |
| --- | --- |
| BF-PSSM (2) | 0.980 |
| Profile (5,7.5) | 0.980 |
| SVM-ngram-LSA | 0.939 |
| LA-eig (β = 0.5) | 0.925 |
| VBKC | 0.924 |
| SVM-BALSA | 0.917 |
| SVM-RQA | 0.912 |
| *SVM-PCD (0.99)* | *0.906* |
| *SVM-PCD (0.999)* | *0.902* |
| *SVM-PCD (All)* | *0.902* |
| SVM-Pairwise | 0.896 |
| SVM-LA | 0.887 |
| (5,1)- Mismatch SVM | 0.872 |
| PSI-BLAST | 0.675 |
